# Experimental Human Cell and Tissue Models of Pemphigus

**DOI:** 10.1155/2010/143871

**Published:** 2010-05-26

**Authors:** Gerda van der Wier, Hendri H. Pas, Marcel F. Jonkman

**Affiliations:** Center for Blistering Diseases, Department of Dermatology, University Medical Center Groningen, University of Groningen, Hanzeplein 1, P.O. Box 30001, 9700 RB Groningen, The Netherlands

## Abstract

Pemphigus is a chronic mucocutaneous autoimmune bullous disease that is characterized by loss of cell-cell contact in skin and/or mucous membranes. Past research has successfully identified desmosomes as immunological targets and has demonstrated that acantholysis is initiated through direct binding of IgG. The exact mechanisms of acantholysis, however, are still missing. Experimental model systems have contributed considerably to today's knowledge and are still a favourite tool of research. In this paper we will describe to what extent human cell and tissue models represent the in vivo situation, for example, organ cultures of human skin, keratinocyte cultures, and human skin grafted on mice and, furthermore, how suitable they are to study the pathogenesis of pemphigus. Organ cultures closely mimic the architecture of the epidermis but are less suitable to answer posed biochemical questions. Cultured keratinocyte monolayers are convenient in this respect, but their desmosomal make-up in terms of adhesion molecules does not exactly reflect the in vivo situation. Reconstituted skin is a relatively new model that approaches organ culture. In models of human skin grafted on mice, acantholysis can be studied in actual human skin but now with all the advantages of an animal model.

## 1. Introduction

Pemphigus is a chronic mucocutaneous autoimmune bullous disease, characterized by the presence of autoantibodies against the desmosomal cadherins, desmoglein 1 (Dsg1), and/or desmoglein 3 (Dsg3). There are two main forms of pemphigus: pemphigus foliaceus (PF) and pemphigus vulgaris (PV). PF presents as superficial blistering of the skin and the presence of autoantibodies against Dsg1. In the case of mucosal dominant PV, patients have suprabasal blistering of the mucous membranes and auto-antibodies against Dsg3 only. Patients with mucocutaneous PV have suprabasal blistering of both the skin and the mucous membranes, in combination with autoantibodies against both Dsg1 and 3.

Since the discovery by Beutner and Jordon in the sixties, who demonstrated by indirect immunofluorescence (IIF) microscopy that sera of pemphigus vulgaris patients contained IgG antibodies directed against a substance on the surface of keratinocytes [1], investigators have tried to answer an intriguing question: how do these antibodies cause acantholysis in skin? In the nineties, Mahoney et al. presented their theories on steric hindrance and desmoglein compensation [2] as an explanation for acantholysis. Recently, researchers are also focusing on other putative mechanisms for example, cell signalling [3, 4], apoptosis [5], desmosome assembly and disassembly [6], and endocytosis [7].

Although the exact steps in the process of acantholysis in pemphigus are still not clear, research herein has considerably benefitted from experimental models, for example, mouse models and in vitro models. Unlike the animal models, the in vitro models have been used to study the effector-phase of pemphigus and not its cell-mediated immune regulation. In this paper we will discuss the in vitro models and focus on human cell and tissue models. These models comprise organ cultures of human skin, cultured human monolayer keratinocytes, reconstituted skin, and human skin grafted on mice. We will discuss how well these human cell and tissue models represent the in vivo situation in human skin and their suitability to study the pathogenesis of pemphigus.

## 2. Organ Cultures of Human Skin

**Figure 1:**
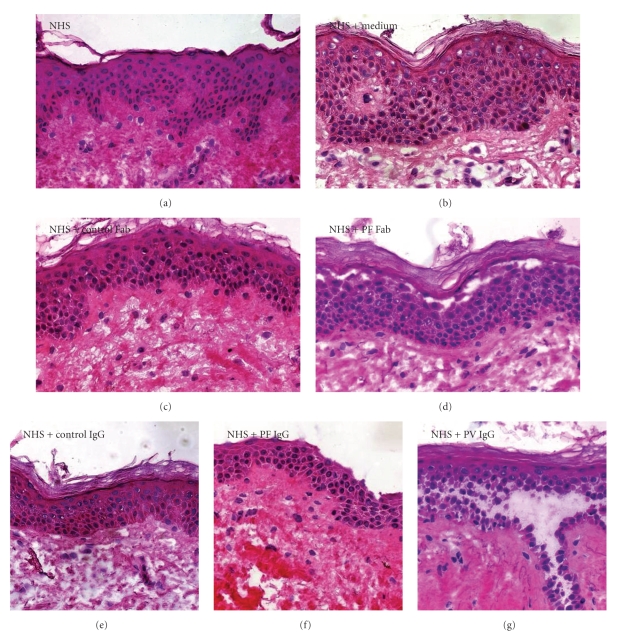
Incubation of normal human skin with pemphigus IgG or Fab fragments leads to suprabasal or subcorneal acantholytic blistering. (a) Normal human skin (NHS) before incubation. Incubation of NHS for 24 hours in (b) medium only or in medium with added (c) control Fab fragments or (e) control IgG leads to limited spongiosis of the epidermis. Incubation of NHS in medium with added (d) PF Fab fragments and (f) PF IgG induces a subcorneal split. Incubation of NHS in medium with (g) PV IgG induces suprabasal acantholysis.

**Figure 2:**
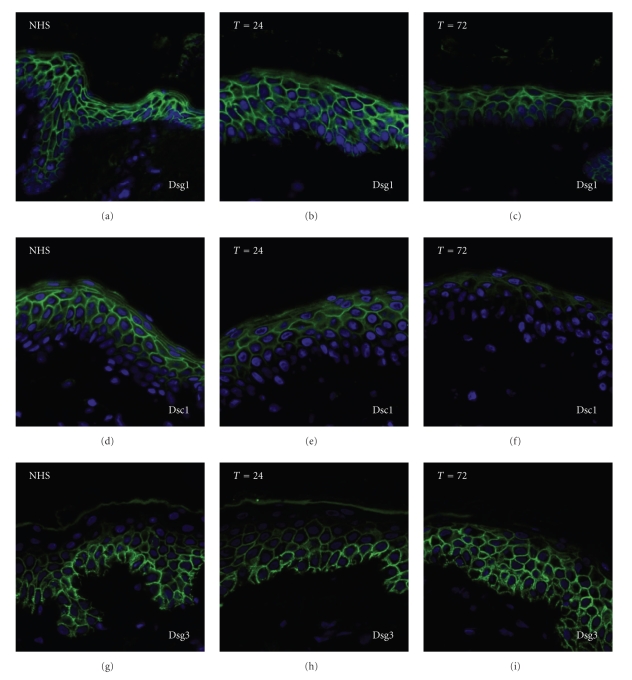
Shift in expression of Dsg1, Dsc1, and Dsg3 in submerged skin cultures after more than 24 hours. (a) Dsg1 is expressed throughout all the layers of NHS. (b) The expression of Dsg1 by skin incubated in medium for 24 hours is comparable to that of NHS. (c) After incubation in medium for 72 hours, Dsg1 expression is reduced. (d) Dsc1 is expressed in the upper layers of the epidermis. (e) The expression of Dsc1 by skin incubated in medium for 24 hours is comparable to that of NHS. (f) After incubation in medium for 72 hours, Dsc1 expression is reduced. (g) Dsg3 is expressed in the basal and suprabasal layers of the epidermis in NHS. (h) The expression of Dsg3 after incubation in medium for 24 hours is comparable to that of NHS. (i) After incubation in medium for 72 hours, Dsg3 is also expressed in the upper layers of the epidermis.

Michel and Ko were among the first who successfully produced acantholysis in vitro by using an organ culture model [8]. They described a relatively simple and reproducible method based on the work of Sarkany et al. [9]. Michel et al. placed a skin explant on lens paper which floated on the surface of liquid that contained crude pemphigus serum. Since then, more research groups have used this organ culture model to study pemphigus [10–18]. We ourselves have recently performed experiments using an organ culture model with air-liquid interface in which a biopsy of normal human skin is not floated on lens paper but instead placed on a transwell such that the bottom of the biopsy contacts the solution containing IgG (Figure 1). In a second approach we submerged biopsies in solution. This enabled culturing more biopsies in one and the same volume of medium with added pemphigus IgG or Fab fragments. Biopsies can be easily harvested at any time and processed for light microscopy, immunofluorescence, or electron microscopy. Although submerged culturing induces shifts in the expression of the different cadherins, for example, substantial loss of Dsg1 and desmocollin 1 (Dsc1) with increased expression of Dsg3 in higher cell layers, this only manifests after prolonged culturing, and their expression remains comparable to normal human skin when the experiments are limited to 24 hours (Figure 2).

Michel and Ko incubated normal human skin with undiluted sera from pemphigus patients. Direct immunofluorescence (DIF) showed intercellular staining of IgG. Light microscopy showed a split after 24-hours incubation. Unfortunately, this first attempt was not as successful as had been hoped and both PF and PV sera induced a suprabasal split [8]. Later investigators, however, did succeed in producing correct subcorneal splits in normal human skin with PF IgG [16]. In our own organ culture model subcorneal acantholysis can be induced not only by PF IgG but also by PF Fab fragments (Figure 1(f)). 

Next to whole serum, Michel and Ko also performed incubations with heated serum in order to inactivate complement [8]. Heated serum also led to acantholysis, which showed that the pathogenesis of pemphigus was not complement dependent [8, 19, 20]. The demonstration that Fab fragments of pemphigus IgG also induce acantholysis confirmed the concept that complement fixation was not a necessary step in this disease [21]. Acantholysis thus is independent of IgG subclass. 

Hu et al. studied the effects of pemphigus IgG incubation on normal human skin by electron microscopy [22]. After 12-hours incubation the first changes, for example, intercellular widening, were seen. After 24 hours the intercellular widening had progressed, and dissolution of the desmosomes became visible. Also desmosome remnants could be seen on the surfaces of the keratinocytes; the tonofilaments had retracted from the cell periphery and were concentrated in a perinuclear position. After 72 hours a suprabasal split and widening between the basal cells (a row of tombstones) were seen [22]. Most of the observations described by Hu et al. are comparable to those seen in pemphigus patient skin, but whether or not the observed retraction of tonofilaments in this organ culture model is comparable to the in vivo situation remains a matter of debate. Unlike others [23], we ourselves did not observe this retraction of tonofilaments in pemphigus patient skin [24].

The human organ culture model has been very valuable in obtaining information on the mechanisms of acantholysis and, moreover, has also been used to test old and new therapeutic drugs for pemphigus, for example, hydrocortisone [13], dapsone [13], methylprednisolone [17], and protease inhibitors [15]. Although most popular in the eighties, it is still used today, often in combination with other models [16, 25–28]. The major advantage of skin explants remains that it is actual human skin with correct architecture of all epidermis layers. Layer-specific changes in morphology or protein localization can easily be studied by light microscopy, immunofluorescence, or electron microscopy. However, explants are less suitable to answer biochemical questions concerning molecular pathways, that is, the activation of receptor molecules or changes in phosphorylation state of pathway intermediates. In contrast to cultured cells, cells in the skin explant reside in layers of varied differentiation that most likely respond differently to external stimuli. Aside from this, cultured cells will instantaneously make contact with the added IgG, while in organ cultures the IgG must diffuse into the epidermis and will not reach all cells simultaneously. Therefore, more easily manageable culture models are the preferred models for biochemical and molecular biological research on acantholysis. 

## 3. Keratinocyte Cultures

A year after the first publication on the organ culture model, Schiltz et al. incubated human keratinocytes with pemphigus IgG [29]. The results of these experiments suggested that binding of pemphigus antibodies to the keratinocytes initiates a series of events which result in the release or activation of hydrolytic enzymes by the keratinocytes with subsequent autolysis and acantholysis. This made it clear that keratinocyte cultures could serve as a model for acantholysis. Various sources of cells are now being used, with most researchers using normal human epidermal keratinocytes (NHEKs) [30–32]. These keratinocytes are often derived from neonatal foreskin [6, 7, 28, 33–37] but can also be obtained from surgical excised skin [38]. HaCaT cells, a non-tumorigenic human keratinocyte cell line, are also popular [16, 39–43]. Less commonly used is the squamous cell carcinoma (SCC) cell line DJM-1 [35, 44].

Cultured keratinocytes are mostly used as monolayers or alternatively as reconstituted skin. Keratinocytes grown in low calcium medium will proliferate until confluent and then become growth arrested. In high-calcium medium (1.2 mM or higher) cells will differentiate, form desmosomes, and stratify [45]. For reconstituted skin, it is a requisite to culture the keratinocytes on a dermal equivalent [46]. Varying the calcium concentration provides a tool to induce and study desmosome assembly and disassembly [6, 45]. Whether cultured keratinocytes are a reliable model, to study pemphigus pathogenesis, be it in the form of monolayers or reconstituted skin, will depend mainly on their ability to form mature desmosomes with correct make-up of cadherins and associated molecules. The expression and localization of the pemphigus antigens and other desmosomal adhesion molecules in cultured cells, therefore, became an early subject of research.

By IIF staining with patient sera it was shown that the PV antigen is expressed in human epidermal monolayers when cultured under high-calcium conditions, but at the same time these monolayers lack the PF antigen [47]. 

Low-calcium cultured monolayer cells do not express Dsg1 [47, 48] and Dsg2 [48], while high-calcium cultured monolayers express Dsg1 [47]. As shown by immunoblot, Dsg1 is detectable after 1 to 6 days of culturing [48], but the expression levels appear to be low [33]. As keratinocytes become stratified, Dsg1 expression increases and can be detected on the plasma membrane of stratified cells in a membrane-bound pattern [47]. The Dsg2 expression in high-calcium cultured monolayers is only positive after 5-6 days as shown by immunoblot [48]. Some groups report that the immunoblot does not show expression of Dsg3 by low-calcium cultured monolayers [48]. Dsg3 was detected in the cytoplasm of cells grown under low-calcium conditions while the protein is translocated to the plasma membrane when cultured under high-calcium conditions [6]. Staining of desmocollin 3 (Dsc3) in NHEKs cultured under low-calcium conditions shows a diffuse cytoplasmic and a focal desmosomal pattern, but comparable to Dsg3 the desmosomal staining intensifies after raising the calcium concentration [6]. 

HaCaT cells are capable of expressing Dsg1, Dsg2, and Dsg3 [43, 48], and similar to normal keratinocytes, Dsg1 expression is induced by high levels of calcium [48].

Most tested SCC cell lines have weak or focal intercellular expression of PV antigens and expression of PF antigens in localized areas [47]. Denning et al. tested several SCC cell lines and showed by immunoblot Dsg2 and Dsg3 expression by these cells when cultured in normal or high-calcium media [48]. Aoyama and Kitajima used the DJM-1 cell line and showed expression of Dsg1 and Dsg3 when cultured in high calcium [49]. 

From the information summarized in Table 1, we can conclude that NHEKs have limitations as an experimental model for pemphigus since these cells do not express significant amounts of Dsg1. Consequently, these experimental systems are not suitable to study acantholysis in PF and mucocutaneous PV. HaCaT cells and DJM-1 cells (Table 1), which express Dsg1 in monolayers, might be more appropriate model systems. It must be taken into account, however, that these cells might express Dsg2 [50] that is not present in most skin areas affected by pemphigus. Despite all drawbacks, monolayers have contributed much to our knowledge on acantholysis and have been at the basis of new ideas and insights. An elegant practical example of their use is the in vitro keratinocyte dissociation assay that can quantify the anti-Dsg3 acantholytic effects of patient IgG [7, 32, 33, 42, 44, 51–53]. After incubation of monolayers with IgG, dispase is used to release the cell sheet from the culture dish. This sheet is then subjected to fierce mechanical stress by means of pumping in and out of a syringe. The resulting number of cell fragments is a quantification of the acantholytic effect of the IgG [54]. An illustration of just how important the cadherin composition of the desmosomes is becomes apparent when HaCaT cells are used in the same assay and no fragments are formed. This is likely due to the high Dsg2 expression [54].

Keratinocytes cultured in reconstituted skin will differentiate and stratify. Therefore both PV and PF antigens are expressed in reconstituted skin [47, 55–57]. By culturing keratinocytes air-exposed on a dermal equivalent, it is possible to reconstruct a multilayered epidermis [46, 58]. The morphology of this reconstituted skin can be compared to that of epidermis in vivo [46, 58, 59]. Ultrastructural assessment of a skin equivalent showed mature desmosome formation [46, 59]. Unfortunately, the expression of the desmosomal proteins, the cadherin antigens, and the formation of desmosomes in these skin equivalents are not well documented. DIF or IIF of desmosomes showed intercellular staining, but in contrast to human skin, there is also strong staining at the top level or cornified layer [58, 59]. Few researchers used reconstituted skin as an in vitro model to study the pathogenesis of pemphigus [60–62].

## 4. Human Skin Grafted on Mice

As mentioned in the introduction, mouse models are often used in pemphigus research next to the human in vitro models. By using mouse models, however, pemphigus is induced in murine skin which might differ in its function from human skin. By grafting human skin on mice, acantholysis can be studied in human skin while at the same time providing the researcher with the advantages of a mouse model [63]. There is only limited experience with these mouse models in pemphigus. Zillikens et al. grafted full-thickness human skin onto the back of SCID mice [64]. PF and PV IgG were injected in the dermis of the graft. Histopathologic findings and DIF of the grafted human skin were comparable to histopathologic findings and DIF in PF and PV patient skin. Others used reconstituted skin grafted onto SCID mice, and subcorneal blistering was induced by injection of PF IgG [27]. These graft models therefore seem very promising.

## 5. Model Comparison

When studying a human disease, a model is required that approaches the in vivo human situation as closely as possible. Studying the pathogenesis of pemphigus in patients unfortunately has its limitations. For ethical reasons, biopsies cannot be taken too often making it impossible to in detail follow the time course of disease development. Mouse models have given great insight into the disease, but mice are not completely comparable to humans, so some questions remain that will have to be addressed in human models. The human in vitro models described in this paper all have their advantages and disadvantages. Therefore no single model may be preferred, but different models may be used in a complementary fashion. Organ cultures and skin equivalents have the advantage that they are most comparable to human skin in terms of desmoglein expression and mature desmosomes. Acantholysis can be evaluated easily with light microscopy, immunofluorescence or electron microscopy. To study pathways and to follow the fate of individual molecules in a narrow time frame in terms of expression level, shifts in localization, phosphorylation, or molecular interaction, easily manageable culture models are favoured. Cell lines should be chosen such that they reflect the skin situation as closely as possible. As discussed before they are suitable to study aspects of acantholysis in PV but not PF for the simple reason that no cells so far have been cultured that express Dgs1 in absence of Dsg3. The mechanism of pemphigus acantholysis has been studied for the past forty years and has taught us which molecules are involved and that acantholysis occurs in the absence of inflammation mediators. How desmosomes split and what molecular pathways lead to acantholysis is still being debated. The use of different experimental models is required to investigate the patho-mechanism. 

## Figures and Tables

**Table 1:** Expression of desmosomal components by monolayers composed of different cell types cultured in low- or high-calcium medium. −: negative, ±: weak positive, +: positive.

	Low calcium				High calcium			
Cell type	Dsg1	Dsg2	Dsg3	Dsc3	Dsg1	Dsg2	Dsg3	Dsc3
NHEK	−	−	−	+	±	+	+	+
HaCaT	−	+	+		+	+	+	
SCC	−	−	−		+	+	+	

## Abbreviations

Dsg: Desmoglein
PF: Pemphigus foliaceus
PV: Pemphigus vulgaris
IIF: Indirect immunofluorescence
IgG: Immunoglobulin G
Fab: Fragment antigen-binding
Dsc: Desmocollin
DIF: Direct immunofluorescence.
